# Ankylosing Spondylitis: A Trade Off of HLA-B27, ERAP, and Pathogen Interconnections? Focus on Sardinia

**DOI:** 10.3389/fimmu.2019.00035

**Published:** 2019-01-25

**Authors:** Fabiana Paladini, Maria Teresa Fiorillo, Valentina Tedeschi, Alberto Cauli, Alessandro Mathieu, Rosa Sorrentino

**Affiliations:** ^1^Department of Biology and Biotechnology “Charles Darwin”, Sapienza University, Rome, Italy; ^2^Department of Medical Sciences and Public Health, Chair of Rheumatology and Rheumatology Unit, University and AOU of Cagliari, Cagliari, Italy

**Keywords:** HLA-B27 alleles, ankylosing spondylitis, ERAP1 and 2, autoimmunity, malaria

## Abstract

The frequency of HLA-B27 in patients with Ankylosing Spondylitis (AS) is over 85%. There are more than 170 recognized HLA-B27 alleles but the majority of them is not sufficiently represented for genetic association studies. So far only two alleles, the HLA-B^*^2706 in Asia and the HLA-B^*^2709 in Sardinia, have not been found to be associated with AS. The highly homogenous genetic structure of the Sardinian population has favored the search of relevant variants for disease-association studies. Moreover, malaria, once endemic in the island, has been shown to have contributed to shape the native population genome affecting the relative allele frequency of relevant genes. In Sardinia, the prevalence of HLA-B^*^2709, which differs from the strongly AS-associated B^*^2705 prototype for one amino acid (His/Asp116) in the F pocket of the peptide binding groove, is around 20% of all HLA-B27 alleles. We have previously hypothesized that malaria could have contributed to the establishment of this allele in Sardinia. Based on our recent findings, in this perspective article we speculate that the *E*ndoplasmic *R*eticulum *A*mino *P*eptidases, ERAP1 and 2, associated with AS and involved in antigen presentation, underwent co-selection by malaria. These genes, besides shaping the immunopeptidome of HLA-class I molecules, have other biological functions that could also be involved in the immunosurveillance against malaria.

## Introduction

HLA-B27 and Ankylosing Spondylitis (AS) is a paradigmatic example of association between the HLA and an immuno-mediated disease (1, 2). Yet, the molecular basis of this association remains unclear. There are several reasons for this failure: the complex genetics of the disease that lacks conclusive animal models; the site of lesions, the enthesis, hard to explore, as well as the lack of specific markers of inflammation (3–5). It has been shown that inflammatory bowel diseases (IBD) and AS frequently co-occur with 5–10% of AS cases having clinical IBD and approximately 70% having subclinical bowel inflammation (6, 7). Moreover, there is a genetic overlap between the two diseases, although HLA-B27 remains a hallmark of AS and its role, although still undisclosed, appears to be prevalent and specific (8, 9).

With the advances in the molecular techniques, more than 170 recognized HLA-B27 alleles (http://www.ebi.ac.uk/cgi-bin/ipd/imgt/hla/allele.cgi) have been identified. However, the majority of them is not sufficiently represented to enable association studies (10). So far only two alleles, the HLA-B^*^2706 in Asia and the HLA-B^*^2709 in Sardinia, have been shown to be absent or rarely present in patients with AS (11–13). Structural and functional differences distinguish the B^*^2706 and B^*^2709 alleles from their closest pair, the B^*^2704 and B^*^2705, respectively (11, 14). Dissecting such differences can therefore give a clue to the molecular basis of this impressive association.

## The HLA-B^*^2709: A Sardinian Allele With a Neutral Effect on AS

The tale of the HLA-B^*^2709 has its origin from studies aimed to characterize, at subtyping level, the HLA-B27-restriction of autoreactive, cytotoxic γδ T cells (15). Surprisingly, these cells killed B-LCLs from HLA-B27 positive members of the proband family but not unrelated HLA-B^*^2705 positive B-LCLs. cDNA sequencing disclosed a single amino acid variance in the binding groove of the HLA-B27 molecules (Asp/His 116) that made highly specific this recognition. Noteworthy, this novel allele, named B^*^2709, was frequent enough to enable association studies in Continental Italy. Most interesting, this allele showed the highest frequency in Sardinia, a Mediterranean island of about 1.6 × 10^6^ inhabitants, where it represents 20% of all HLA-B27 subtypes (13). So far, we have analyzed a total of 50 HLA-B^*^2709-positive subjects from Sardinia and only one was affected by AS. However, this patient carried also another AS-risk allele, the B^*^1403 (16). Therefore, to the best of our knowledge, at least in Sardinia the B^*^2709 allele does not confer susceptibility to AS. Although the B^*^2709 is harbored by a different HLA haplotype compared to the B^*^2705, there is little evidence supporting this as an explanation for this lack of association (17, 18). Moreover, the observation that another allele, the B^*^2706, more frequent in Asia and differing from the common B^*^2704 for two amino acids Asp/His 114 and Tyr/Asp 116 (11), both located in the peptide binding groove, gave strength to the hypothesis of the “arthritogenic” peptide/s (19). This predicts the existence of a specific HLA-B27-restricted “self immunopeptidome” triggering autoreactive CTLs primarily elicited by cross-reactive microbial peptides. This opened a research vein pointing to analyse the immunopeptidomes eluted from HLA-B27 molecules associated or non-associated with the disease (20). Indeed, although in the course of several decades many structural and functional differences could be observed, nevertheless no clear-cut indications came out from these studies and other scientifically substantiated hypotheses have been proposed (14, 21–23). More recently, a report has singled out 26 peptides preferentially eluted from the HLA-B27 associated alleles, giving a new ground to search for candidate peptides possibly involved in disease pathogenesis (24).

## ERAP1 and ERAP2 Contribute to as Susceptibility

A strong support to the theory that antigen presentation contributes to the disease, came from the finding that the ER-resident aminopeptidases, ERAP1 and ERAP2 showed also an association with AS and other “MHC-I-opathies” (25–29). These intracellular enzymes are pivotal to the generation of 8–10 mer peptides that serve as ligands for HLA-class I molecules by trimming longer (ERAP1) or shorter (ERAP2) peptides as discussed in depth by Lopez de Castro in this same Research Topic (30). However, while ERAP1 appears to act in epistasis with the predisposing HLA-class I molecules, ERAP2 does not (26, 31). Indeed, ERAP2 has been found to confer susceptibility also to other diseases for which a stringent association with HLA-class I has not been established, as in the case of Crohn's Disease. This suggests a role for ERAP2 by mechanisms which go beyond the peptidome shaping.

## HLA Region Undergoes Positive Selection

HLA-class I genes fulfill a crucial role in the defense against intracellular invaders: the more efficient they are, the more likely is the surviving of the species. Therefore, infectious agents represent one of the most powerful selective pressure impacting on the human genome in general and, specifically, on the HLA genes (32, 33). This can generate the potential for disorders that involve phenotype alterations such as, in the case of HLA region, autoimmune diseases. The question whether the risk alleles have undergone positive selection has been highly debated. There are however, molecular genetic data that strongly support this hypothesis (33–38). An example of a locus that appears to have been subject to strong positive selection is the HLA 8.1 ancestral haplotype, a conserved combination of HLA genes present in 15% of Caucasians, that plays a key role in the inflammatory response and protects from infectious diseases. As a counterpart, its presence is associated with an increased risk toward several autoimmune disorders (39, 40).

In this context, HLA-B27 positive subjects appear to display a more effective immunosurveillance against some viruses such as HIV, hepatitis C and EBV either because able to present relevant epitopes and/or by contributing to the inflammatory microenvironment needed for an effective response to occur (14). As in the case of HLA 8.1 haplotype, the HLA-B27 could confer as trade off, a higher predisposition to autoimmune/autoinflammatory diseases such as AS.

## The Case of HLA-B27

Although a high number of HLA-B27 alleles have been described, only for few of them a worldwide distribution and a functional impact has been documented: i.e. the ancestral and strongly AS-associated B^*^2705, the B^*^2702 and B^*^2707 more frequent in the Middle East, the B^*^2704 and B^*^2706 in the East, the B^*^2703 in Africa and the B^*^2709 in Sardinia (11, 14).

Most interestingly, there is a descending gradient from north to south of the HLA-B27 allelic distribution. In particular, the ancestral HLA-B^*^2705 covers a wide spectrum of frequencies: from more than 20% in North Europe to < 1% in the sub-Saharan Africa (41, 42). Of note, the distribution of the above mentioned non-B^*^2705 subtypes follows an opposite gradient which is superimposable to that of malaria: more frequent in the area in which malaria was endemic and lower in North Europe.

These observations lead us to postulate that HLA-B^*^2705 allele could have been counter-selected by malaria and that the establishment of the different subtypes has been favored by this environmental pressure (42).

However, negative selection is very difficult to prove. A hint to this theory could come from the analysis of the allelic distribution of other genes, also participating in the shaping of the HLA-B27 peptidome.

## Did the Aminopeptidases Undergo Selection by Malaria? Suggestive Observations

The aminopeptidases ERAP1, ERAP2, and LNPEP (Leucyl-cystinyl aminopeptidase) are intracellular enzymes encoded by polymorphic genes contiguously located on chromosome 5q15. Some of these polymorphisms are functional and have been found associated with AS. In the case of ERAP1, the missense SNP rs30187 has been functionally related to the level of trimming activity and, notably, the allelic variant associated with AS shows a more efficient ERAP1 enzymatic activity (26). ERAP2, that associates with AS in its HLA-B27-negative forms as well (31), shows two main haplotypes in strong Linkage Disequilibrium (LD), HapA and HapB, the latter not expressing ERAP2 due to the balanced polymorphism at SNP rs2248374 that impacts on RNA stability. Therefore, 25% of the population is ERAP2 negative (43). It has been recently proposed that this balanced strong LD might be due to the role that ERAP2 can play in the immune evasion of trophoblasts (44). The analysis of its distribution in AS patients and healthy subjects has indicated that the absence of ERAP2 is protective for AS as well as for other inflammatory diseases, although the reasons for this protection are far from being elucidated (31, 45). Interestingly, the full expression of ERAP2 as observed in HapA homozygous individuals, appears to play a role in protecting from HIV infection, most probably modulating antigen presentation (46). This is a further indication that autoimmunity can be the downside of an effective immune response. In this context, a recent report has indicated CD8^+^ T cells as key players in the development of cerebral malaria, a life-threatening complication of the *P. falciparum* infection. These T cells have a two-faced role: in the parasite clearance from blood and liver and in the induction of neuroinflammation. This is apparently due to CCDC88B, a risk locus for several auto-inflammatory conditions among which IBD and psoriasis, both of which showing a genetic overlap with AS (47). Although this gene has not been found associated with AS, nevertheless other genes involved in the CD8^+^ T cells differentiation and activity do have (26).

More recently, other ERAP2 functional polymorphisms have been described. In particular, our group has shown that the minor allele G at SNP rs75862629 in the promoter region of ERAP2 couples a lower expression of ERAP2 with a higher expression of ERAP1 (48). Interestingly, this finding reveals for the first time that the transcription of the two genes is interlinked. There are several possible explanations for this observation: i.e. a direct competition of the two genes for the same transcription factor/s or a steric hindrance or even an indirect effect due to intermediates. Since the worldwide frequency of this minor allele ranges between 5 and 15%, it will be most important to test its association with the “MHC-I-opathies,” in the different populations.

**Figure 1:**
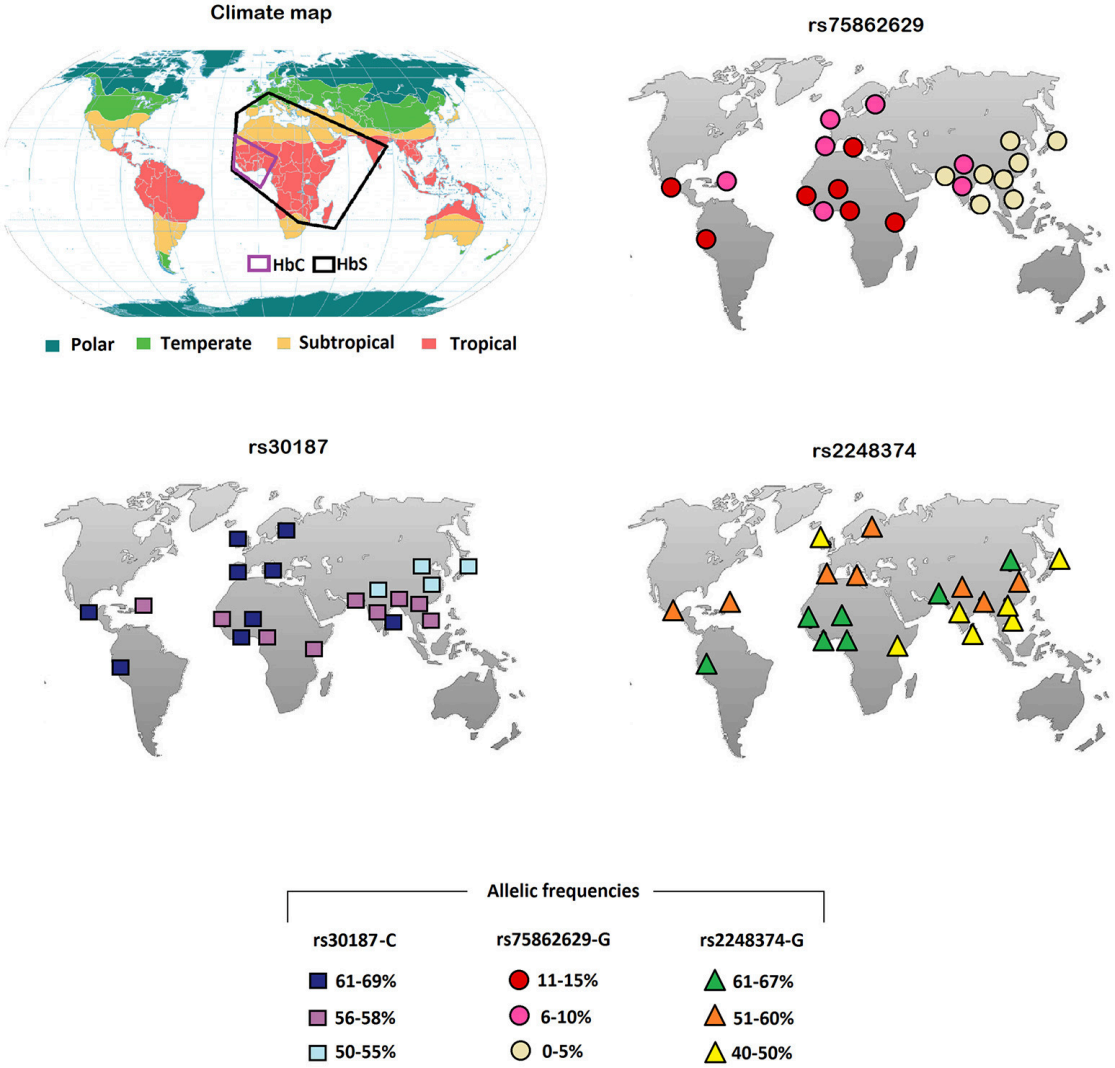
Climate plan-sphere and the world allelic frequencies (https://www.ncbi.nlm.nih.gov/variation/tools/1000genomes/) of the ERAP rs30187 C variant (bottom left), or of the ERAP2 rs75862629 G (top right) and rs2248374 G (bottom right). The black and purple boxes indicate, respectively, the regions where HbS and HbC hemoglobin variants are more frequent.

Here, we have analyzed the worldwide distribution of some SNPs in the aminopeptidase genes. In particular, we report the frequencies derived from the 1,000 genomes (https://www.ncbi.nlm.nih.gov/variation/tools/1000genomes/) of the missense rs30187 in the ERAP1 coding region, as well as the distribution of rs2248374 and rs75862629 in the ERAP2 gene (Table 1; Figure 1). Although the data are based only on some ethnic groups, we noticed an interesting distribution of the ERAP2 polymorphisms: the G alleles at both rs75862629 and rs2248374, which co-segregate respectively, with a lower or null expression of ERAP2, were more frequent in the equatorial regions. This is reminiscent of the malaria distribution as well as of the occurrence of the HLA-B27 subtypes.

**Table 1:** Allele frequencies of C, G, G, and A variants at rs30187, rs75862629, rs2248374, and rs2303138 respectively, in the populations analyzed in the 1,000 Genomes Project.

**Population**	**Allelic frequency (%)**			
	**rs30187**	**rs75862629**	**rs2248374**	**rs2303138**
	**C**	**G**	**G**	**A**
(YRI) Yoruba in Ibadan, Nigeria	58.3	14.8	60.2	5
(LWK) Luhya in Webuye, Kenya	60.1	13.2	41.9	1.5
(GWD) Gambian in Western Gambia	57.5	13.7	67.3	8.8
(MSL) Mende in Sierra Leone	62.4	9.4	64.1	5.3
(ESN) Esan in Nigeria	64.1	13.1	61.6	6.1
(MXL) Mexican Ancestry from Los Angeles, USA	60.9	11.7	52.3	2.3
(PUR) Puerto Ricans from Puerto Rico	57.2	10.1	57.2	7.7
(PEL) Peruvians from Lima, Peru	67.1	13.5	63.5	2.1
(CHB) Han Chinese in Beijing, China	50.5	1	62.1	44.7
(JPT) Japanese in Tokyo, Japan	54.8	0.5	48.6	36.1
(CHS) Southern Han Chinese	51.4	0	57.6	44.3
(CDX) Chinese Dai in Xishuangbanna, China	57	0	47.9	39.3
(KHV) Kinh in Ho Chi Minh City, Vietnam	60.1	0	43.9	33.3
(FIN) Finnish in Finland	68.7	6.6	54.5	7.6
(GBR) British in England and Scotland	63.6	7.7	44	5.5
(IBS) Iberian population in Spain	62.6	9.4	53.7	7.5
(TSI) Toscani in Italy	62.6	13.5	56.5	4.7
Pakistan(PJL) Punjabi from Lahore, Pakistan	60.4	4.2	60.4	16.2
(GIH) Gujarati Indian from Houston, Texas	54.8	7.8	58.7	19.9
(BEB) Bengali from Bangladesh	57	3.5	51.7	20.3
(STU) Sri Lankan Tamil from the UK	63.2	3.5	49.5	18.6
(ITU) Indian Telugu from the UK	59.8	9.8	43.6	10.8

In Sardinia, the malaria has been endemic since the old age up until the '50s and has favored the establishment of genetic variants more efficient in fighting the adverse effects of Plasmodium infection (49). This was not without a price and a higher presence of diseases such as thalassemia or G6PD deficiency is a common trait in Sardinia (50). More recently, a robust evidence in the selection of variants conferring susceptibility to some autoimmune diseases has also been provided (51, 52).

As for ERAP2, the frequency in Sardinia of the rs75862629 MAF is among the highest (about 14%). These observations seem to suggest that ERAP2 could have been a target of selection and, more precisely, those alleles determining a lower expression of ERAP2 appear to be favored in the areas where the *P. falciparum* has been endemic. Is this because a lower expression of ERAP2 confers some protection from the most severe form of malaria?

There are some observations that could give support to this speculation: (1) the co-segregation with HLA-B27 subtypes whose occurrence has been already proposed as consequence of a selection by *P. falciparum* (49); (2) the multifaceted nature of this enzyme: ERAP2, besides being involved in antigen presentation, has a role in the regulation of blood pressure as well. Interestingly, a lower activity of ERAP2 has been correlated with a basal diastolic higher pressure (53), whose implication as a protective factor against malaria is becoming an increasingly accredited hypothesis. In this context, a lower expression of ERAP2 has been found in the first trimester of pregnancy in women that will develop pre-eclampsia (PE), a disease that is the main cause of maternal and fetal morbidity and has a strong genetic background (54). Recent studies in Australian/New Zealand and Norwegian populations have shown that one of the two possible ERAP2 haplotypes is associated with PE susceptibility (55). The same variations have been also associated with increased risk of hypertensive disorders in pregnancy in African American population (56) but not in the Chilean population. Of note, this population has also a different distribution of the ERAP2 SNPs which are not in complete LD as in the other populations (57).

It is therefore conceivable that malaria is the factor that has favored the establishment of polymorphisms correlating with a lower expression of ERAP2. The allele G at rs2248374 that determines the ERAP2 mRNA non-sense mediated decay, has a frequency higher than 60% in the equatorial Africa and, remarkably, the minor allele at rs75862629, also correlating with a lower ERAP2 transcription, reaches its highest frequency in the same regions (Table 1; Figure 1). In this context, it is interesting to note that the worldwide distribution of abnormal variants of hemoglobin (HbS, HbC) selected by malaria (58) shows a good overlap with that of G variant at rs75862629 (Figure 1). This supports the hypothesis that hypertension, in particular when co-occurring with erythrocyte variations that reduce Plasmodium proliferation, can play a protective role against Plasmodium infection.

Of note, in East Asia, where the frequency of AS is much lower than in the western hemisphere (59), haplotype combinations in the region where ERAP1, ERAP2, and LNPEP map, are different with a prevalence of ERAP2 variants that favor protein expression. To which extent this might be due to genetic drift, ERAP2 selection, or variants in LD with ERAP2, it remains to be established. It is suggestive, however, that in Asia, *P. vivax* rather than *P. falciparum*, is the main cause of malaria (60). This observation also might suggest that, where ERAP1/ERAP2/LNPEP genomic region has undergone a selective pressure from malaria, it may even have operated at both systemic and immunological level. In fact, like ERAP1 and ERAP2, LNPEP regulates the renin–angiotensin system and is involved in peptide trimming, as well (61). In addition, LNPEP is able to activate the NF-κB pathway (62). Noteworthy, in Asia, where ERAP2 displays a minor degree of polymorphism, LNPEP variants have been described to correlate with the onset of autoinflammatory phenomena, as well as with susceptibility to hypertension: i.e., the A variant in LNPEP rs2303138, in East Asia (40% in Asia, 5–6% in the rest of the world) (Table 1) is in LD with the productive allele A in ERAP2 rs2248374, but correlates with a lower expression of LNPEP (63). This, as consequence, determines in the serum a higher level of its target, the Angiotensin II, which could limit the erythrocyte invasion by Plasmodium avoiding the complications of cerebral malaria (64).

## Conclusions

In conclusion, functional variants of the three aminopeptidases ERAP1, ERAP2, and LNPEP show a worldwide distribution compatible with a selective pressure by malaria. Some of these variants co-occur with HLA-B27 subtypes. In particular, in Sardinia the distribution of the ERAP2 rs75862629 minor allele correlates with the presence of the HLA-B^*^2709 subtype. It will be interesting to investigate if this pair shows any functional interaction and if this has any correlation with AS susceptibility/protection.

## Author Contributions

The ideas in this perspective article were jointly conceived by FP, MF, and RS, who wrote the manuscript. All authors contributed to the discussion of the draft and made the final corrections.

### Conflict of Interest Statement

The authors declare that the research was conducted in the absence of any commercial or financial relationships that could be construed as a potential conflict of interest.
